# Unexpected extrapyramidal symptoms and pulmonary aspergillosis in exertional heatstroke with fulminant liver failure: a case report

**DOI:** 10.1186/s13256-016-1184-0

**Published:** 2017-02-10

**Authors:** Jie Jiao, Feihu Zhou, Hongjun Kang, Chao Liu, Mengmeng Yang, Jie Hu

**Affiliations:** 1 Critical Care Medicine, Hainan Branch of Chinese PLA General Hospital, Haitangwan District, Sanyan, Hainan Province 572000 China; 2 Critical Care Medicine, Chinese PLA General Hospital, 28th Fuxing Road, Haidian District, Beijing, 100853 China

**Keywords:** Case report, Heatstroke, Liver failure, Extrapyramidal symptoms, Pulmonary aspergillosis, Therapeutic plasma exchange

## Abstract

**Background:**

Exertional heatstroke is a life-threatening condition with high mortality because of the rapid progress of multiple organ dysfunction syndrome even if aggressive treatments are initiated rapidly. Mild to moderate hepatic injury is common in exertional heatstroke, while fulminant liver failure is rare. Extrapyramidal symptoms and pulmonary aspergillosis secondary to liver failure induced by exertional heatstroke have never been reported in prior cases.

**Case presentation:**

A 25-year-old Han Chinese man presented with exertional heatstroke with fulminant liver failure, subsequent pulmonary aspergillosis, and extrapyramidal symptoms. Moreover, he also presented with coma, rhabdomyolysis, acute kidney injury, and disseminated intravascular coagulation. He recovered under conservative treatment including therapeutic plasma exchange plus continuous veno-venous hemofiltration, fluid resuscitation, antibiotics, and other support therapy.

**Conclusions:**

Therapeutic plasma exchange plus continuous veno-venous hemofiltration could be effective for patients with heatstroke who suffer liver failure and other organ failure. Patients with liver failure are at high risk for pulmonary aspergillosis. Movement disorder in these patients might be extrapyramidal symptoms induced by consistent low level of cholinesterase resulted from hepatic injury besides brain injury.

## Background

Heatstroke is clinically diagnosed as a severe elevation in body temperature, central nervous system (CNS) dysfunction, and a history of environmental heat exposure or vigorous physical exertion [1]. Classic heatstroke primarily occurs in immunocompromised individuals during annual heat waves. Exertional heatstroke (EHS) is observed in individuals who are highly motivated to perform strenuous physical activity in hot weather [1]. To date, the treatment for heatstroke is aggressive cooling, fluid resuscitation, and other organ support therapy. Despite these efforts, EHS mortality is high and is usually associated with multi-organ failure, especially hepatic injury and coagulopathy [2–4]. However, mild to moderate hepatic injury is common in EHS, while fulminant liver failure has been reported only in about 5 % of patients [5]. Emerging evidence suggests that multi-organ damage is a consequence of direct thermal injury to the tissues, coagulopathies, and development of a systemic inflammatory response syndrome (SIRS) that is stimulated by endotoxin, cytokines, and other immune modulators. In this report, we describe the case of a 25-year-old man who presented with EHS with fulminant liver failure, subsequent pulmonary aspergillosis, and extrapyramidal symptoms.

## Case presentation

A previously healthy 25-year-old Han Chinese man started feeling dizzy after running 5 km on a rainy night with an ambient temperature of about 30 °C. His surface temperature was 41.2 °C (axillary) and he was taken to a nearby hospital. On presentation at the hospital, he was unconscious, with hyperpyrexia of 40.2 °C, tachycardia of 129 beats/min, and blood pressure of 122/76 mmHg. Orotracheal intubation was performed because of the deterioration of his mental status. His initial blood tests demonstrated elevated transaminases, bilirubin, creatine kinase and creatinine, thrombocytopenia, and deteriorating coagulation. The patient was diagnosed as EHS and multiple organ dysfunction syndrome (MODS). In spite of cooling, aggressive fluid resuscitation and blood purification, hepatic and renal dysfunction continuously deteriorated. Therefore, the patient was transferred to our critical care medical unit 5 days later (day 5). On admission, our patient was unconscious (no sedation, with a Glasgow Coma Score (GCS) of 5) with mechanical ventilation. His temperature was 38.2 °C (axillary), heart rate (HR) was 50 beats/min, respiratory rate (RR) was 15/min, and his blood pressure (BP) was 103/70 mmHg (norepinephrine 0.2ug/kg/min). Laboratory data revealed worsened hepatic and renal function, deterioration of coagulation parameters, and markers of infection. A cranial and abdominal computed tomography (CT) scan showed no abnormal findings; only the pulmonary CT scan demonstrated little consolidation (Fig. 2a). An electrocardiogram (ECG) revealed sinus bradycardia, prolonged QT interval and abnormal ST-T.

**Fig. 1:**
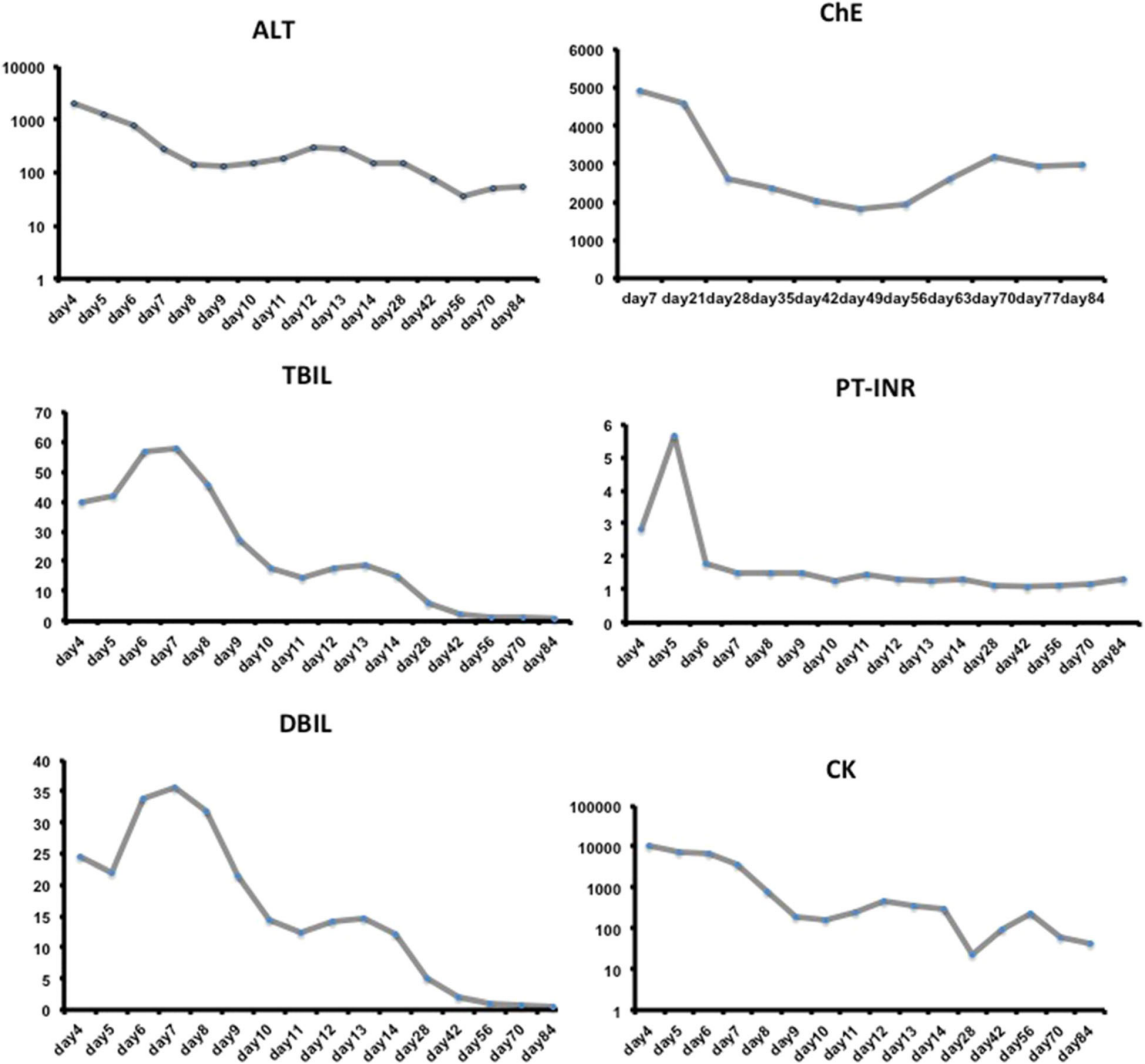
Blood test results, including alanine transaminase (*ALT*), total bilirubin (*TBIL*), direct bilirubin (*DBIL*), prothrombin time-international normalized ratio (*PT-INR*), creatine kinase (*CK*), and cholinesterase (*ChE*)

These biochemical disturbances reflected acute liver failure (ALF). On admission (day 5), the results of his blood biochemistry exhibited high transaminases, 1204.2 U/L alanine aminotransferase (ALT) (normal < 40 U/L), 363.9 U/L aspartate aminotransferase (AST) (normal < 40U/L), significantly elevated total bilirubin (TBIL) 47.0 mg/dL (normal < 1.16 mg/dL), and prothrombin time-international normalized ratio (PT-INR) of 5.65, decreased prothrombin activity (PTA) of 14 % (80 % < normal < 130 %), fibrinogen of 1.16 g/L (2 g/L < normal < 4 g/L) and cholinesterase of 4134U/L (ChE, 4650U/L < normal < 12220U/L) (Fig. 1), and normal blood ammonia of 100.7 μg/dL (27.2 μg/dL < normal < 102 μg/dL). To exclude other causes for ALF, virus serological tests were performed. There were no positive findings for acute or chronic hepatitis A, B, C, E or human immunodeficiency virus (HIV). Also, acute infection with Epstein-Barr virus (EBV) and cytomegalovirus (CMV) was ruled out. The test results for autoimmune antibodies (ASMA, AMA, ANA, ANCA, ENA) were negative. A past history review showed no recent mushroom and herbal products ingestion, and no medication history. Moreover, our patient already met the ALF criteria (TBIL 107.5umol/L and INR 3.1) outside our hospital when his hemodynamic status was stable on day 2, which could rule out hypoxic hepatitis. In addition, an abdominal ultrasound and CT scan did not exhibit evidence of dilated bile ducts and portal embolism. Because of worsening hyperbilirubinemia, therapeutic plasma exchange (TPE) was performed in the subsequent week, even though its role in ALF remained unclear. With the fifth session, it was noted that our patient was more responsive and started to open his eyes spontaneously. His laboratory parameters were also showing an improvement in liver function with resolving coagulopathy (Fig. 1). TPE was discontinued after six sessions and his TBIL decreased from then. It is worth mentioning that his blood ammonia remained normal during the whole process.

Our patient also suffered rhabdomyolysis with a creatine kinase level of 10121U/L (CK, 2U/L < normal < 200U/L) with normal urine output and a mild increase of creatinine because of adequate fluid resuscitation. With the purpose of avoiding rhabdomyolysis-induced acute kidney injury and alleviating SIRS, our patient received continuous veno-venous hemofiltration (CVVH) every day from day 5 to day 10. With the fifth session, it was noted that his CK level (Fig. 1) declined gradually and his creatinine level was normal.

**Fig. 2:**
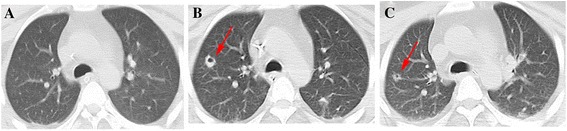
Computed tomography images of lung. **a** Computed tomography scan on day 4 showing no abnormality. **b** Computed tomography scan on day 23 showing emerging nodules with cavity. **c** Computed tomography scan on day 38 showing narrowing nodules. *Red arrows*, cavity

Our patient presented persistent fever accompanied with an increase of white blood cells (WBC) and neutrophils. On admission, his WBC count was 24,430/μL, N% 88 %, procalcitonin 2.11 ng/ml (normal < 0.5 ng/ml). A sputum culture (day 14) showed *Aspergillus* and *Acinetobacter baumannii*. Considering liver failure could dampen host immune function, caspofungin was added. Subsequently, a pulmonary CT scan (day 23) demonstrated emerging nodules with cavity (Fig. 2b). Thus pulmonary aspergillosis was diagnosed even though the galactomannan (GM) test was negative. A tracheotomy was performed on day 29 not only to favor pulmonary infection control but also to safely use sedative medication due to his undefined movement disorder. With antifungal therapy and recovery of hepatic function, his pulmonary infection improved and a pulmonary CT scan (day 38) demonstrated nodules (Fig. 2c). He was successfully weaned from mechanical ventilation on day 34.

**Fig. 3:**
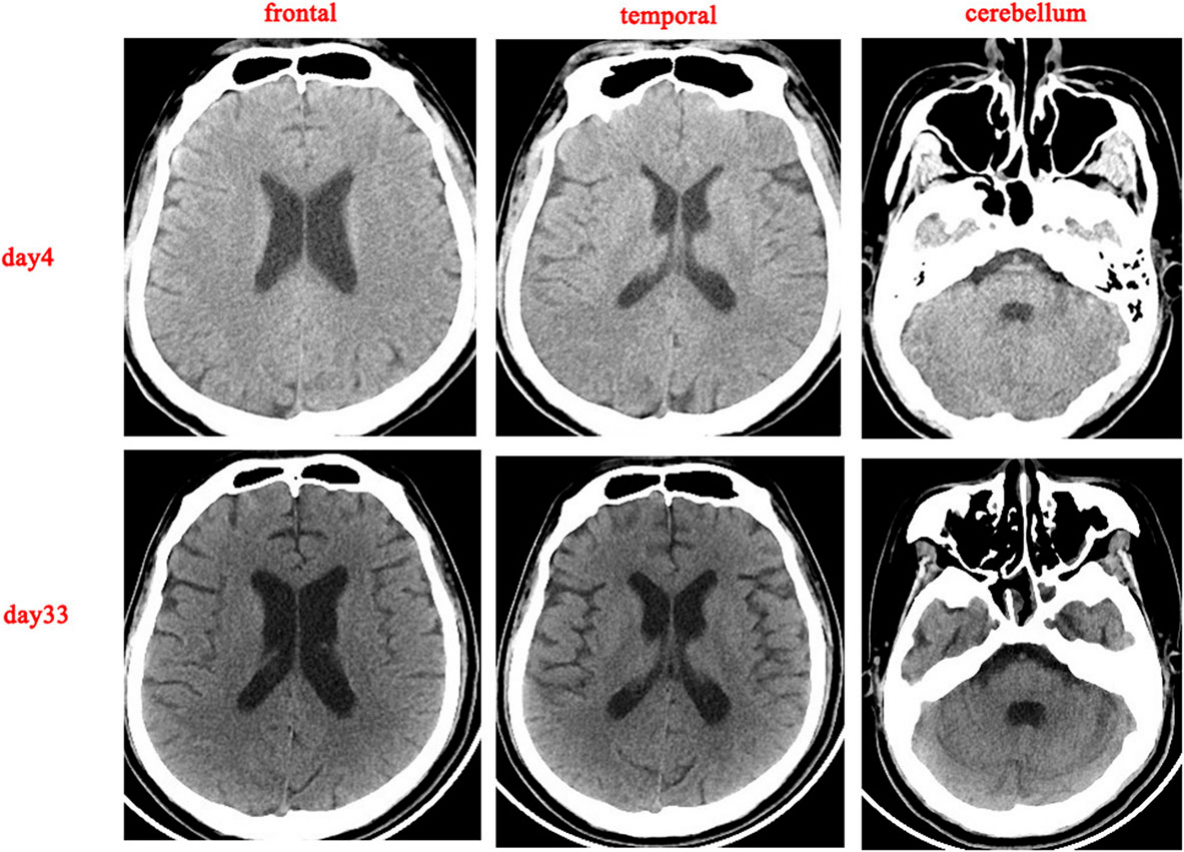
Computed tomography images of the brain showing atrophy of the temporal and frontal lobe and the cerebellum, comparing the computed tomography scan on day 4 to the computed tomography scan on day 33

On day10, sedation was stopped to evaluate mental status. The patient regained eyes open spontaneously but experienced dystonia-like involuntary movements of head and mouth but not extremities. At first, it was diagnosed as seizures secondary to heatstroke related CNS dysfunction. However, we found that promethazine could aggravate involuntary movements while diazepam could temporarily mitigate the symptoms, which indicated that this might not be the case. Electroencephalogram (day 52) showed no abnormal findings and cranial CT (day 4, 33) demonstrated atrophy of frontal lobe (Fig. 3a), temporal lobe (Fig. 3b) and cerebellum (Fig. 3c), which might damage the conduction path of extrapyramidal system. What’s more, liver failure induced consistent low ChE (Fig. 1), probably leading cholinergic and dopaminergic function disorder. Thus, we believed that those involuntary movements were extrapyramidal symptoms. Benzhexol hydrochloride plus scopolamine therapy were initiated (day 53) and diazepam managed to mitigate head and mouth movement disorder even the patient was not sedated. As the recovery of hepatic function and increase of ChE, the dose of benzhexol hydrochloride decreased.

Finally, at the time of discharge from the ICU (day 93), our patient presented mental retardation, mild extrapyramidal symptoms, and his laboratory tests had returned to normal values except for minor ALT elevation.

## Discussion

In this case, we described a young patient with severe EHS that was mainly complicated with fulminant liver failure and subsequent pulmonary aspergillosis and extrapyramidal symptoms, as well as coma, rhabdomyolysis, acute kidney injury, and disseminated intravascular coagulation. The success in treating this EHS case suggested that therapeutic plasma exchange (TPE) and CVVH might be effective in EHS cases with fulminant liver failure and other organ failure.

Heatstroke is clinically diagnosed as a severe elevation in body temperature that occurs in the presence of central nervous system (CNS) dysfunction and a history of environmental heat exposure or vigorous physical exertion [1]. A retrospective study demonstrated that major organ dysfunction involved in heat-related illness included neurological (100 %), renal (57 %), hepatic (34 %), and coagulation abnormalities (26 %) [6]. Approximately 5 % of EHS patients experienced fulminant liver failure, which might be fatal [7]. However, according to Kilian [8] and Garcin [9], acute liver failure (AHF) is relatively frequent during heatstroke and the incidence might be much higher than is usually thought, and hypophosphatemia on admission could predict occurrence of ALF during heatstroke [9]. In this case, TBIL was as high as 57.7 mg/dL (Fig. 1a, day 7) and PT was as high as 52.2 seconds (day 5), which fulfilled accepted London criteria [10] for emergency liver transplantation (PT longer than 50 seconds, bilirubin higher than 17.5 mg/dL, and non-A, non-B hepatitis). The reason that we still chose conservative therapy such as TPE other than orthotopic liver transplantation (OLT) was as follows: (1) the pathogenesis of liver failure might be direct thermo-injury to hepatic cells and overwhelming SIRS, which could be conservatively managed and spontaneously recover [5, 11–13]; (2) the outcome of liver transplantation in heatstroke in the previous case studies was controversial [14, 15] but the immunosuppressor might have aggravated sepsis-induced organ failure in the mid or late phase in heatstroke [16]; (3) the coagulopathy could be improved by transfusion or medication while the blood ammonia levels were as high as 115.3 μg/dL on day 7 (27.2 μg/dL < normal < 102 μg/dL, male). After TPE six times, the TBIL level decreased to 14.97 mg/dL (day 14) and the hepatic function recovered spontaneously.

TPE can be used for thrombotic microangiopathy and acute liver failure [17]. To date, only one study was randomized and controlled in patients with ALF to demonstrate a conclusive improvement with conservative therapies [18]. Even whether TPE could treat ALF was controversial, although some case reports have proved that TPE might be effective in EHS with ALF. Raj *et al*. demonstrated that the addition of therapeutic plasma exchange plus continuous veno-venous hemodiafiltration (CVVHDF) resulted in a reversal of the inflammatory process and recovery from multi-organ failure in an 11-year-old obese boy who suffered EHS with rhabdomyolysis and concurrent renal, pulmonary, and liver failure [13]. Miura *et al*. also demonstrated that a previously healthy 38-year-old Japanese man who developed EHS following a long-distance run and presented with fulminant hepatic failure (FHF) accompanied by a life-threatening flare-up of rhabdomyolysis. Plasma exchange and hemodiafiltration enabled the patient to survive FHF [19]. In our case, like the young man in the former case report [13], our patient finally recovered from ALF, rhabdomyolysis, and concurrent renal and pulmonary failure after TPE and CVVH therapy, which indicated that TPE plus blood purification might be effective in heatstroke with ALF, rhabdomyolysis, and other concurrent organ failure.

Encephalopathy is a universal manifestation of heatstroke, occurring abruptly, and can be severe in most of the cases. Commonly, encephalopathy improves dramatically by cooling in 70–90 % of heatstroke patients [20]. Those who did not regain consciousness by cooling or initial therapies always developed seizures or focal motor deficit [20]. The most conspicuous histological damage to the CNS of heatstroke patients includes progressive degeneration of neurons in the cerebellum and cerebral cortex with congestion, edema, and microhemorrhages at autopsy [1]. In this case, a cranial CT scan demonstrated atrophy of the frontal lobe (Fig. 3a), temporal lobe (Fig. 3b), and cerebellum (Fig. 3c), which could explain his mental retardation and extrapyramidal symptoms. In addition, benzhexol hydrochloride, a kind of anticholinergic drug, managed to mitigate our patient’s movement disorder, which confirmed the onset of extrapyramidal symptoms. To date, there has been no report on heatstroke-induced extrapyramidal symptoms. Theoretically, consistent low ChE (Fig. 1) might exaggerate cholinergic and dopaminergic function disorder in the context of frontal atrophy. Moreover, as hepatic function recovered and ChE level increased, the dose of benzhexol hydrochloride decreased. Thus, liver failure and decreasing ChE might be involved in extrapyramidal symptoms in this case.

SIRS plays an important role in heatstroke pathogenesis as sepsis. In that, early death might be due to overwhelming inflammation while late death might be due to persistent immunosuppression and irreversible infection [21]. Tarek reported a 25-year-old man with heatstroke presenting with severe rhabdomyolysis and massive hepatic necrosis, who died 41 days after the liver transplantation of systemic infection due to fungal and bacterial sepsis [16]. In this regard, prior case reports have documented a high prevalence of *Candida* spp. infections in heatstroke victims [22]. Invasive pulmonary aspergillosis (IPA) is a rapid, progressive, fatal disease that occurs mostly in immunocompromised patients. It has been previously demonstrated that patients with decompensated liver disease are more prone to pulmonary aspergillosis [23, 24]. In this case, our patient exhibited high fever, elevated WBC and PCT, aspergillus of sputum culture (day14), and emerging nodes on a pulmonary CT scan (day 23), which indicated invasive pulmonary aspergillosis (IPA). Given the ALF and potential acute kidney injury due to rhabdomyolysis, caspofungin rather than voriconazole or amphotericin B (there was no liposomal amphotericin in our hospital) was chosen as the first-line treatment. Fortunately, caspofungin was able to control the above symptoms and thus was used as monotherapy in this patient with IPA.

## Conclusions

In conclusion, we for the first time report an exertional heatstroke case with liver failure complicated with unexpected extrapyramidal symptoms and pulmonary aspergillosis. Unlike other cases, the movement disorders were induced by consistent low ChE level, which can exaggerate cholinergic and dopaminergic function disorder in the context of frontal atrophy. Thus, as to EHS patients with multiple organ dysfunction characterized by fulminant liver failure, appropriate supportive therapy, such as TPE and CVVH rather than liver transplantation could be justified in the early stage and substantially reduce the mortality and thus was strongly recommended. Anticholinergic drugs, such as benzhexol hydrochloride or scopolamine, could mitigate movement disorders. Further clinical research needs to weigh the risk of fulminant liver failure in EHS.

## Abbreviations

ALT: alanine aminotransferase
AST: aspartate aminotransferase
ChE: cholinesterase
CK: creatine kinase
CMV: cytomegalovirus
CNS: central nervous system
CT: computed tomography
CVVH: continuous veno-venous hemofiltration
EBV: Epstein-Barr virus
EHS: exertional heatstroke
GCS: Glasgow Coma Score
GM: galactomannan
HIV: human immunodeficiency virus
IPA: invasive pulmonary aspergillosis
MODS: multiple organ dysfunction syndrome
PTA: prothrombin activity
PT-INR: prothrombin time-international normalized ratio
SIRS: systemic inflammatory response syndrome
TBIL: total bilirubin
TPE: therapeutic plasma exchange
WBC: white blood cells
